# Supplementary material for: Effect of Post-Hatch Heat-Treatment in Heat-Stressed Transylvanian Naked Neck Chicken
Source: Animals (Basel). 2021 May 27;11(6):1575. doi: 10.3390/ani11061575 (PMC8227715; doi:10.3390/ani11061575)
Supplement: Supplementary file 1 [file animals-11-01575-s001.zip › animals-1179250-supplementary.pdf]

Supplementary Materials

Table S1:

A:

**Number of hens and roosters used at sperm quality and egg production analysis**					
**Sperm analysis**		**Egg production**			
HS	HTHS	HS		HTHS	
male	male	male	female	male	female
10	10	3	31	3	31

A: The number of chickens in ***Experiment I.*** The egg production and sperm analysis experiments in case of heat treated and heat stressed (HTHS) and the control which was only heat stressed (HS) group.

B1:

**Number of chicks and adults used at RNA expression experiments (pooled RNA samples)**							
**Chick**				**Adult**			
Ctrl		HT		Ctrl		HT	
male	female	male	female	male	female	male	female
13	2	9	6	5	4	8	4

B2:

**Number of chicks and adults used at RNA expression experiments (individual RNA samples)**							
**Chick**				**Adult**			
Ctrl		HT		Ctrl		HT	
male	female	male	female	male	female	male	female
4	2	4	4	4	4	4	4

B: Summarized the pooled (**B1**) and individual (**B2**) heat-treated (HT) and control (Ctrl**)** RNA samples from ***Experiment II***.

Table S2:

Gene	ID	Primer set (5' - 3')	Product size	Tm (°C)
**GAPDH**[a]	NM_204305.1	**F:** 5'-CTTTGGCATTGTGGAGGGTC-3'<br>**R:** 5'-ACGCTGGGATGATGTTCTGG-3'	128	60
**ß ACTIN**[a]	NM_205518.1	**F:** 5'-ACCTGAGCGCAAGTACTCTGTCT-3'<br>**R:** 5'-CATCGTACTCCTGCTTGCTGAT-3'	95	60
**HSF1**[a]	L06098.1	**F:** 5'-CAGGGAAGCAGTTGGTTCACTACACG-3'<br>**R:** 5'-CCTTGGGTTTGGGTTGCTCAGTC-3'	192	60
**HSF2**[a]	NM_001167764.1	**F:** 5'-CGCTGCTCGCATTCCT-3'<br>**R:** 5'-TGTGGCCTCACTTGCTTCT-3'	194	60
**HSF3**[a]	XM_420166.3	**F:** 5'-TCCACCTCTCCTCTCGGAAG-3'<br>**R:** 5'-CAACAGGACTGAGGAGCAGG-3'	71	60
**HSF4**[a]	NM_001172374.1	**F:** 5'-TGCCAGCCTTCCTAACCAAG-3'<br>**R:** 5'-TGGTGCCATTCGTACTCCAG-3'	84	60
**HSPA2 (Hsp70)**[a]	NM_001006685	**F:** 5'-CGTCAGTGCTGTGGACAAGAGTA-3'<br>**R:** 5'-CCTATCTCTGTTGGCTTCATCCT-3'	145	60
**HSP90AA1 (Hsp90)**[a]	NM_001109785.1	**F:** 5'-GAGTTTGACTGACCCGAGCA-3'<br>**R:** 5'-TCCCTATGCCGGTATCCACA-3'	107	60
**CHD1-Z**[b]	NC_006127.4	**F:** 5'-TATCGTCAGTTTCCTTTTCAGGT-3'<br>**R:** 5'-CCTTTT ATTGATCCATCAAGCCT-3'	461	54
**CHD1-W**[b]	NC_006126.4	**F:** 5'-TATCGTCAGTTTCCTTTTCAGGT-3'<br>**R:** 5'-CCTTTT ATTGATCCATCAAGCCT-3'	322	53.2

S2: Information about the used primer pairs and target genes. In case of heat-shock markers (a) the applied primers were chose from the article of Xie et al. [11]. For sex determination, (b) the applied *CHD1* primer-pair was chosen from the paper of Lee et al. [53]. In every case during the real-time PCR examination the *GAPDH* and *ß ACTIN* were the housekeeping genes.

Figure S1:

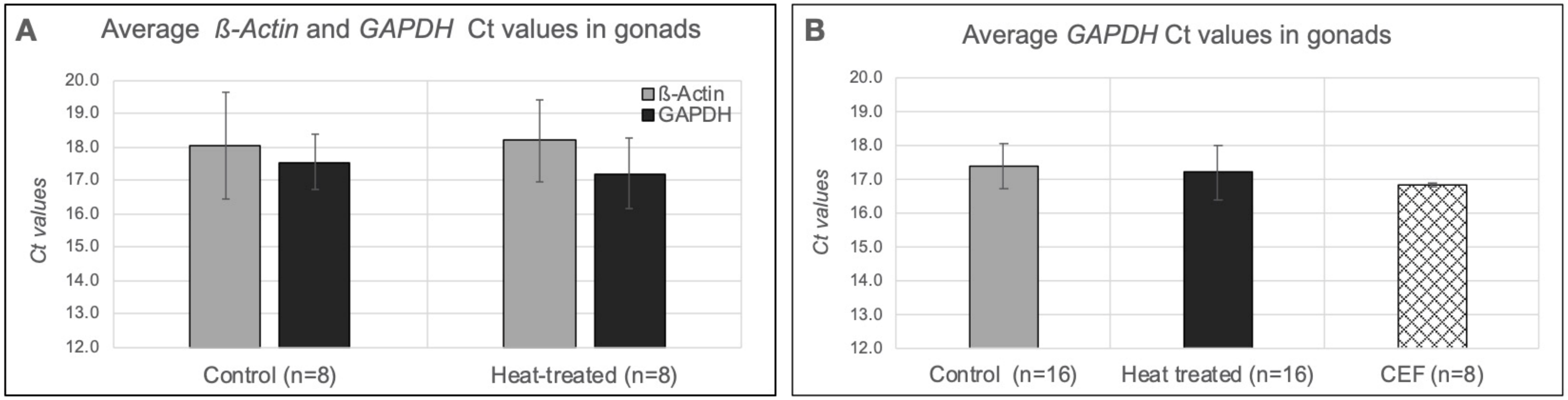

A.: The figure presents the expression of two housekeeping genes (*GAPDH* and *ß-Actin*). We compared the average Ct values of *GAPDH* and *ß-Actin* in control and heat-treated samples. We found lower Ct values in the case of *GAPDH*, and the standard deviation were higher using *ß-Actin*.

B.: There was no significant difference between the control and heat-treated samples (p= 0.523) comparing the average *GAPDH* Ct values. We used chicken embryonic fibroblast as the reference sample

Figure S2:

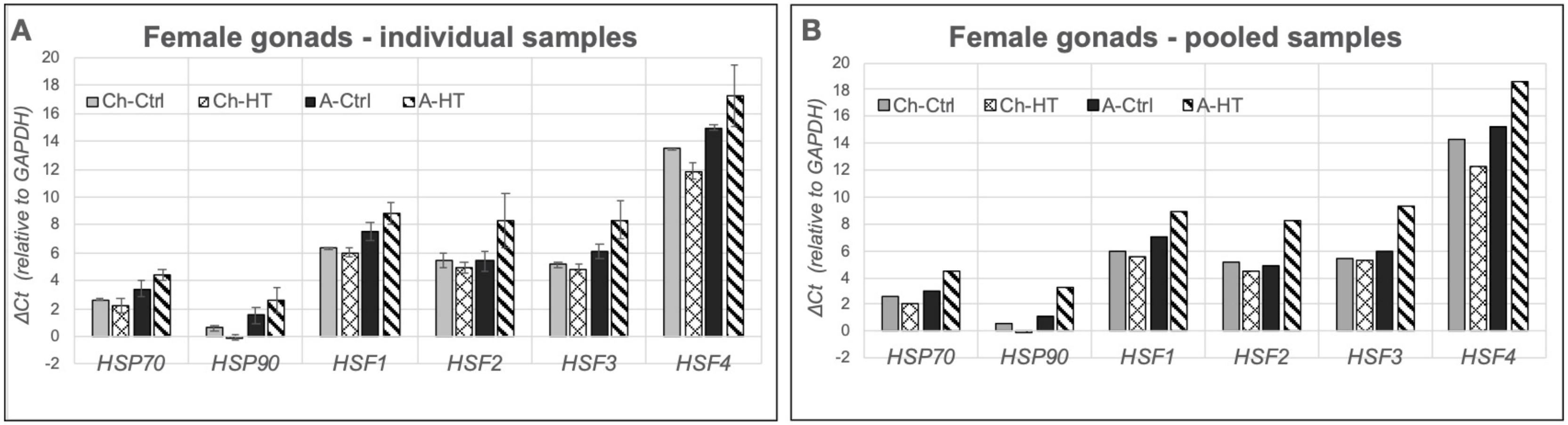

This figure represents the similarity between the individual and pooled delta Ct values measured in female gonads.

A: Average values and standard deviations of individual samples are demonstrated.

B: Delta Ct values measured in pooled samples.

(Ch-Ctrl: Chick-control; Ch-HT: Chick-heat-treated; A-Ctrl: Adult-control; A-HT: Adult-heat-treated)

References

11. Xie, J.; Tang, L.; Lu, L.; Zhang, L.; Xi, L.; Liu, H.-C.; Odle, J.; Luo, X. Differential Expression of Heat Shock Transcription Factors and Heat Shock Proteins after Acute and Chronic Heat Stress in Laying Chickens (Gallus gallus). *PLoS ONE* **2014**, *9*, e102204, doi:10.1371/journal.pone.0102204.
53. Lee, J.C.-I.; Tsai, L.-C.; Hwa, P.-Y.; Chan, C.-L.; Huang, A.; Chin, S.-C.; Wang, L.-C.; Lin, J.-T.; Linacre, A.; Hsieh, H.-M. A novel strategy for avian species and gender identification using the CHD gene. *Mol. Cell. Probes* **2010**, *24*, 27–31, doi:10.1016/j.mcp.2009.08.003.
